# C-reactive protein identifies patients at risk of postpancreatectomy hemorrhage

**DOI:** 10.1007/s00423-022-02440-9

**Published:** 2022-03-20

**Authors:** C. Vilhav, J. B. Fagman, E. Holmberg, P. Naredi, C. Engström

**Affiliations:** Department of Surgery, Institute of Clinical Sciences, Sahlgrenska Academy at the University of Gothenburg, Sahlgrenska University Hospital, Gothenburg, Sweden

**Keywords:** Pancreatic cancer, Postpancreatectomy hemorrhage, C-reactive protein, Pancreaticoduodenectomy, Postoperative pancreatic fistula, Pancreatic surgery

## Abstract

**Background:**

Postpancreatectomy hemorrhage grade C (PPH C) is a dreaded complication after pancreaticoduodenectomy (PD) with high mortality rate. Concurrent risk factors for PPH C have been difficult to recognize. Connection between postoperative pancreatic fistulas (POPF) and PPH C is well known, but POPF is often unknown prior to the PPH. The aim of this retrospective study was to define potential predictive factors for PPH C.

**Methods:**

Retrospectively, 517 patients who underwent PD between 2003 and 2018 were included in the study. Twenty-three patients with PPH C were identified, and a matched control group of 92 patients was randomly selected. Preoperative data (body mass index, cardiovascular disease, history of abdominal surgery, biliary stent, C-reactive protein (CRP), ASA-score), perioperative data (bleeding, pancreatic anastomosis, operation time), and postoperative data (CRP, drain amylase, POPF, biliary fistula) were analyzed as potential predictors of PPH C.

**Results:**

High postoperative CRP (median 140 mg/L on day 5 or 6) correlated with the development of PPH C (*p* < 0.05). Postoperative drain amylase levels were not clinically relevant for occurrence of PPH C. Grade C POPF or biliary leak was observed in the majority of the PPH C patients, but the leaking anastomoses were not detected before the bleeding started.

**Discussion:**

High postoperative CRP levels are related to an increased risk of PPH C.

## Introduction

Pancreaticoduodenectomy (PD) is an extensive surgical procedure with high morbidity and non-negligible mortality, even in high-volume centers. Late postpancreatectomy hemorrhage (PPH) has an incidence of 3–16% and is one of the most dreaded complications [1, 2]. The International Study Group of Pancreatic Surgery (ISGPS) has categorized PPH depending on time of onset, bleeding site, severity, and clinical impact [3] (Table 1). PPH grade C (PPH C) is by definition a life-threatening complication, with a mortality rate as high as 50% [4, 5].

**Table 1:** International Study Group of Pancreatic Surgery classification of postpancreatectomy hemorrhage

Grade	Time of onset, location, severity, and clinical impact of bleeding		Clinical condition	Diagnostic consequence	Therapeutic consequence
A	Early intra- or extra-luminal mild		Well	Observation, blood count, USG, and, if necessary, CT	No
B	Early intra- or extra-luminal severe	Late intra- or extra-luminal, mild	Often well/intermediate very rarely life-threatening	Observation, blood count, USG, angiography, CT endoscopy	Transfusion of fluid/blood, ICU, therapeutic embolization relaparotomy for early PPH
C		Late intra- or extra-luminal, severe	Severely impaired life-threatening	Angiography, CT endoscopy	Localization of bleeding, angiography and embolization, (endoscopy) or relaparotomy, ICU

Early bleeding: < 24 h postoperatively. *PPH* postpancreatectomy hemorrhage, *USG* ultrasonography, *CT* computed tomography, *ICU* intensive care unit

Pancreatic or biliary juices in the surgical field are thought to erode the walls of the vessels, causing pseudoaneurysms, which eventually might lead to potentially lethal bleeding [2, 6, 7]. The mechanisms are not completely known. The pathogenesis of hemorrhagic complications during pancreatitis is multifactorial and generally associated with inflammation [8]. Inflammation has also been suggested to play an important role in the development of postoperative pancreatic fistula (POPF), though the detailed pathophysiology remains poorly understood [9]. According to the current definition of biochemical leak (BL) and POPF by the ISGPS [10], the fistula is described as grade C if a potentially lethal bleed occurs, which by definition is categorized as PPH C. A strong connection between POPF and PPH has been demonstrated in earlier studies [2, 5, 11]. In the clinical reality, the pancreatic or biliary leakage is often undetected until the bleeding occurs. In this study, we have therefore used the POPF definition to reveal and evaluate leakage of amylase before and after the PPH C, although according to the definition, the real POPF status only can be stated after the patient is rehabilitated.

Abdominal drains are commonly and traditionally used to detect and drain pancreatic or biliary leaks with the extended purpose to avoid PPH. The effectiveness of drains after PD has been questioned, and the benefit has not yet been proven [12]. So far, randomized trials have been discordant and more studies are needed to improve knowledge of the clinical impact of abdominal drains after PD [13, 14]. For other surgical procedures, such as colorectal or gastric resection, drains have been frequently applied, but after being proven ineffective, the use of drains has markedly decreased [15–17].

Sentinel bleeding is a common warning sign before the pseudoaneurysm bleeding becomes very severe, but it can be misinterpreted and not handled properly [18, 19]. The management of PPH C has gradually changed over the years from open surgery attempting to control the bleeding to minimal invasive angiography-guided treatment. The accessibility for angiography has also progressively improved, and interventional radiology has become the standard first-line treatment, though there is no consensus or guidelines regarding the management of PPH C. As the prognosis of PPH C remains poor and no preventive measures have been identified [2, 5], recognizing patients at high risk of developing PPH C is of uttermost clinical importance.

The aim of this study was to define potential preoperative, perioperative, and postoperative predictive factors for PPH C in patients undergoing PD.

## Material and methods

### Patient selection

Patients, who underwent PD between January 2003 and June 2018 at the Sahlgrenska University Hospital, Gothenburg, Sweden, a regional center for pancreatic surgery since 2009 and national center for advanced pancreatic surgery since 2017, were identified using the ICD-10 classification in the medical record. A total of 560 patients were classified as PD (Whipple’s operation); 73 patients were excluded due to contemporary multivisceral operations or transplantations (n:29), arterial resections (n:11), multitrauma operations (n:3), colon resections (n:12), splenectomies (n:10), or other including default encoding (n:8). We chose to exclude major concurrent procedures in order to reduce the number of confounding factors. Both multivisceral and arterial resections are associated with increased mortality and morbidity [20, 21].

Another 64 patients classified as being treated with a total pancreatectomy were re-analyzed. Twenty-three of them were found to be incorrectly classified and were re-classified as PDs. Thus, a total of 510 patients were retrospectively included in the analysis.

The indication for surgery was known or suspected periampullary cancer or precancerous conditions, such as intraductal papillary mucinous neoplasm (IPMN), mucinous cystic neoplasm (MCN), or secondary malignancies. By using our ERAS (enhanced recovery after surgery) register, established in January 2015, patients operated between January 2015 and June 2018 with Clavien-Dindo Grade III or worse were identified. These patients’ medical records were then studied in detail to identify patients with PPH C. To recognize patients with PPH C operated between January 2003 and December 2014, the medical records from discharge and return visits to the surgical outpatient clinic 4 to 6 weeks after the surgery were studied. In total 23 patients with PPH C were identified (Table 3). Five patients with late intraluminal PPH and according to the ISGPS definition severe (more than 3 units packed cells and relaparotomy) hemorrhage were excluded from the PPH C group. In these five patients with intraluminal PPH, the bleeding was controlled by a suture ligature of a bleeding vessel on the pancreatic surface or in the gastroenteroanasomis during a relaparotomy, and the patients recovered quickly after the bleeding had stopped. The five patients were indeed PPH C, but the purpose of this study was to address more complex life-threatening bleedings, extraluminal, with potential underlying anastomosis leakage.

The remaining 487 PD patients were matched against the PPH C patients according to gender, year of operation (± 1 year), and age (± 2 years). Four matched controls were randomly selected for each PPH C patient, a total of 92 patients.

### Surgery and perioperative and postoperative management

The PD was performed as a Whipple procedure in all included patients. The head of the pancreas, the distal part of the stomach, the duodenum, the gallbladder, and the bile duct were removed. The dissection was performed as a standardized procedure at the clinic with electrical scissors and Ligasure™ Impact or Ligasure™ Maryland (Medtronic). The gastroduodenal artery was closed by nonabsorbable suture ligature. The pancreatic remnant reconstruction was performed as a pancreaticojejunostomy (PJ) with duct-to-mucosa until 2010/2011, when there was a gradual shift in the anastomosis technique to pancreaticogastrostomy (PG) with invagination technique. When PG was not possible, as in patients with prior gastric bypass surgery, PJ was performed instead. Perioperatively, two gravidity drains were placed in the abdominal cavity, one towards the hepaticojejunostomy and one towards the pancreatic anastomosis. All patients received preoperative antibiotic prophylaxis. Proton pump inhibitors were given during the first postoperative month. Low molecular weight heparin (LMWH) were routinely administrated to all patients starting the day before surgery and continued until discharge from the hospital. Octreotide was used neither as prophylaxis nor as treatment. A nasogastric tube was routinely inserted, and fasting with gastrointestinal decompression was continued in 3–7 days.

An ERAS program was implemented in January 2015, after which both drains and nasogastric tubes were removed at an earlier time-point. Drains were withdrawn on day 3 instead of day 5 if the drain amylase level was decreasing, < 2400U/L, and the drain fluid serous. The nasogastric tube was removed on day 3 instead of day 7 if the measured volume was < 400 ml.

All patients, even if domiciled in other regions, had a return visit to the Sahlgrenska University hospital at 4 to 6 weeks postoperatively to receive information on the pathology report and for a clinical follow-up.

### Data collection

Preoperative, perioperative, and postoperative data were collected retrospectively from medical records, and the data were saved without personal information for general anonymous analysis.

The potential risk factors for PPH C were divided into three categories: (1) preoperative factors (age, gender, year of surgery, body mass index (BMI), cardiovascular disease, history of abdominal upper gastrointestinal surgery or pancreatitis, preoperative biliary stent, C-reactive protein (CRP) levels, American Society of Anesthesiologists (ASA) score), (2) perioperative factors (amount of bleeding in ml, type of pancreatic anastomosis, operation time), and (3) postoperative factors (BL/POPF before and after bleeding, biliary fistula, and CRP levels). Additional information regarding sentinel bleeding, postoperative day of bleeding and site, pathological examination, angiography, re-operation, 90-day mortality rate, and whether POPF grade C was known before PPH C were also recorded.

Cardiovascular diseases were evaluated by the WHO definition. Biliary fistula was defined as the presence of bile or bile-stained fluid from surgical drains on or after postoperative day 3 [7]. The definition of sentinel bleeding was a minor blood loss via abdominal drains, wound, or the gastrointestinal tract, with an asymptomatic interval until development of hemorrhagic shock [22].

CRP was calculated as the median on postoperative day 2 or 3 (CRP POD 2/3) or postoperative day 5 or 6 (CRP POD 5/6). The number of patients with CRP levels > 180 mg/L was also recorded. Prior to 2015, the days when CRP was measured in the clinic were not standardize, which is why CRP levels were calculated both POD 2/3 and POD 5/6 in this study. After the introduction of the ERAS program in 2015, the CRP levels were always measured on days 1, 3, and 5. The cut-off of 180 mg/L was chosen based on a previous study of pancreas-specific complications after pancreaticoduodenectomy [23].

Preoperative biliary stents were placed by either endoscopic retrograde cholangiopancreatography (ERCP) or percutaneous transhepatic cholangiography (PTC). The median perioperative bleeding volume was measured, and the number of patients with bleeding > 500 ml was calculated separately. The operation time was recorded as the number of patients operated on in < or > 416 min. In the Swedish National Pancreatic Register, the median bleeding at the Sahlgrenska University Hospital Gothenburg was 500 ml in 2017 and 2018; the median operation time was 418 and 416 min, respectively.

### Statistical analysis

The present study is a retrospective matched control study. The statistic method “Power analysis for matched case–control studies” was used to calculate the number of controls to achieve a power of 80%. The controls were matched against the cases according to gender, year of operation (± 1 year), and age (± 2 years). Four matched controls were randomly selected for each case.

Conditional logistic regression was used to calculate odds ratios (ORs) and *p*-values in univariable analyses, and the significant data were then analyzed in a multivariable analysis. Statistical tests were two-sided and *p* < 0.05 considered significant. Data are presented as numbers and percentages or medians and interquartile ranges (IQRs). Statistical analyses were carried out in Stata/IC 15.1 (StataCorp. 2019. Stata: Release 16. Statistical Software. College Station, TX: Stata Corp LLC).

### Ethics

The Regional Ethics Review Board of Gothenburg approved the study (nr: 2019–02,435).

## Results

### Patient characteristics

We identified 23 patients with PPH C: 7 females (30%) and 16 males (70%). The matched control group of 92 patients comprised 28 females (30%) and 64 males (70%). The median age at time of surgery was 68 years in both groups. The pathologists’ examination confirmed twenty-two specimens in the PPH C group as pancreatic and periampullary malignancies and one benign inflammation, pancreatitis (Table 2). In the control group, 77 patients (84%) had pancreatic and periampullary malignancies, 7 (8%) had neuroendocrine or other malignant tumors, and 8 (9%) had benign specimens. There were no significant differences considering the number of malignancies between the groups. Venous resections were performed in two patients (9%) in the PPH C group and 15 patients (16%) in the matched control group and did not constitute a risk factor for PPH C in the subgroup analysis (Table 3).

**Table 2:** Characteristics of patients with postpancreatectomy hemorrhage grade C

Age at operation years	Year of operation	Survival after operation* Days	Pancreatic anastomosis	BL or POPF before PPH	BL or POPF after PPH	Sentinel bleed	POP day of PPH C	Site of bleeding**	Interventional radiology	Emergency operation	Histopathology
60	2004	394	PJ	POPF B	POPF C	Yes	12	Splenic artery	No	Yes	Pancreatic adenocarcinoma T3N1
60	2006	+	PG	POPF B	POPF C	Yes	13	Unknown	No	Yes	Benign, pancreatitis
62	2006	+	PJ	BL	POPF C	Yes	31	Proper hepatic artery	No	Yes	Ampullary adenocarcinoma T3N0
71	2007	23	PJ	BL	POPF C	Yes	21	Unknown	No	Yes	Pancreatic adenocarcinoma T2N1
76	2007	12	PJ	BL	POPF C	Yes	9	Unknown	No	Yes	Pancreatic adenocarcinoma T3N1
67	2009	263	PJ	POPF B	POPF C	No	17	Unknown	No	Yes	Cholangiocarcinoma T3N1
71	2009	+	PJ	POPF B	POPF C	Yes	10	Unknown	No	Yes	Ampullary adenocarcinoma T1N1
61	2011	+	PG	POPF B	POPF C	No	17	Splenic artery	No	Yes	Ampullary adenocarcinoma T3N0
70	2011	+	PG	BL	POPF C	Yes	28	Right hepatic artery	Yes	No	Ampullary adenocarcinoma T3N0
71	2011	408	PJ	No	POPF C	No	12	Common hepatic artery	No	Yes	Pancreatic adenocarcinoma T3N0
57	2012	56	PG	No	POPF C	Yes	10	Proper hepatic artery	Yes	Yes	Ampullary adenocarcinoma T1N0M0
68	2014	17	PG	BL	POPF C	Yes	16	Unknown	No	Yes	Cholangiocarcinoma T3N1
59	2014	10	PG	No	No, infection	No	6	Gastroduodenal artery	No	Yes	Pancreatic adenocarcinoma T4N1
74	2015	14	PG	BL	POPF C	Yes	13	Gastroduodenal artery	Yes	Yes	Pancreatic adenocarcinoma T3N1
77	2015	30	PG	No	POPF C	Yes	23	Hepatic artery	No	Yes	Pancreatic adenocarcinoma T3N1
54	2015	229	PG	No	POPF C	Yes	37	Hepatic artery	Yes	No	Pancreatic adenocarcinoma T3N1
66	2016	171	PG	BL	POPF C	Yes	12	Branches of superior mesenteric artery	Yes	No	Pancreatic adenocarcinoma T3N1
73	2017	583	PG	BL	POPF C	Yes	9	Common hepatic artery	Yes	Yes	Cholangiocarcinoma T3N1
70	2017	359	PG	No	POPF C	No	8	Gastroduodenal artery	Yes	Yes	Pancreatic adenocarcinoma T2N1
77	2017	23	PG	BL	POPF C	Yes	21	Hepatic artery	Yes	No	Ampullary carcinoma in situ TisN0
80	2017	619	PG	BL	No, biliary fistula	Yes	21	Gastroduodenal artery	Yes	No	Duodenal carcinoma T3N1
56	2018	+	PG	BL	POPF C	Yes	16	Splenic artery	Yes	No	Ampullary carcinoma in situ TisN0
63	2018	512	PG	BL	No, biliary fistula	No	34	Common hepatic artery	Yes	No	Pancreatic adenocarcinoma T1N1

^*^ + Indicates if the patient was still alive in January 2021

^**^ Hepatic artery represents common hepatic artery, proper hepatic artery, and the area where the gastroduodenal artery have been suture ligated. In these cases, the angiography could not specify the exact bleeding site, or it was not possible to define the source of the bleeding perioperatively

*PJ* pancreaticojejunostomy, *PG* pancreaticogastrostomy, *BL* biochemical leak, *POPF* postoperative pancreatic fistula, *POP* postoperative

**Table 3:** Univariable conditional logistic regression analysis of predictive factors for postpancreatectomy hemorrhage grade C

Variable	Cases		Controls	Odds ratio (95% CI)	*p*-value
Operation					
Whipple	21 (91%)		77 (84%)	Ref	
Whipple + vein	2 (9%)		15 (16%)	0.45 (0.08–2.34)	0.339
Pancreatic anastomosis					
Pancreaticogastrostomy	16 (70%)		58 (63%)	Ref	
Pancreaticojejunostomy	7 (30%)		34 (37%)	0.38 (0.06–2.44)	0.311
ASA score					
1	7 (30%)		22 (24%)	Ref	
2	10 (43%		51 (55%)	0.59 (0.18–1.87)	0.367
3	6 (26%)		19 (21%)	0.98 (0.24–3.97)	0.975
BMI, kg/m^2^ median	24.6 (22.7–29.7)		23.6 (22.3–26.6)	1.12 (1.01–1.23)	0.026
< 18.5	0 (0%)		5 (5%)	-	
18.5–25	12 (52%)		50 (55%)	Ref	
> 25	11 (48%)		36 (40%)	1.36 (0.50–3.69)	0.543
Cardiovascular disease					
No	5 (22%)		16 (17%)	Ref	
Yes	18 (78%)		76 (83%)	1.34 (0.42–4.28)	0.623
History of upper abdominal surgery					
No	15 (65%)		71 (77%)	Ref	
Yes	8 (35%)		21 (23%)	1.71 (0.67–4.37)	0.261
Preoperative biliary stent					
No	4 (17%)		22 (24%)	Ref	
Yes	19 83%)		70 (76%)	1.50 (0.46–4.89)	0.504
CRP preoperative, mg/L median	7 (3.5–13.5)		7 (3–14.5)	0.99 (0.95–1.04)	0.754
0–20	14 (88%)		53 (83%)	Ref	
> 20	2 (12%)		11 (17%)	0.66 (0.12–3.62)	0.628
CRP day 2/3, mg/L median	210 (160–290)		130 (81–210)	1.14 (1.06–1.22)*	0.001
0–180	8 (35%)		59 (65%)	Ref	
> 180	15 (65%)		32 (35%)	4.00 (1.37–11.7)	0.011
CRP day 5/6, mg/L median	140 (110–180)		80 (52–120)	1.19 (1.09–1.31)*	< 0.001
0–180	17 (74%)		79 (90%)	Ref	
> 180	6 (26%)		9 (10%)	3.41 (1.01–11.5)	0.048
Operation time, min					
0–416	11 (48%)		52 (57%)	Ref	
> 416	12 (52%)		40 (43%)	1.50 (0.55–4.05)	0.425
Bleeding at primary operation, ml median	1600 (500–2000)		1000 (580–1500)	1.03 (0.99–1.08)	0.133
≤ 500	6 (26%)		21 (23%)	Ref	
> 500	17 (74%)		71 (77%)	0.80 (0.25–2.60)	0.716
BL, POPF-B, POPF-C	Before bleeding	After bleeding			
No	6 (26%)	1 (4%)	58 (63%)	Ref. (before bleeding)	
BL	12 (52%)	2 (9%)	24 (26%)	4.05 (1.35–12.1)	0.012
POPF-B	5 (22%)	0 (0%)	4 (4%)	12.6 (2.09–76.2)	0.006
POPF-C	0 (0%)	20 (87%)	0 (0%)		
Data missing	0 (0%)	0 (0%)	6 (7%)		
Biliary fistula					
No	21 (91%)		90 (98%)	Ref	
Yes	2 (9%)		2 (2%)	4.0 (0.56–28.4)	0.166

^*^Per 10 units

Data are presented as *n* (%) or median (IQR) unless otherwise noted. *ASA* American Society of Anesthesiologists, *BMI* body mass index, *ERCP* endoscopic retrograde cholangiopancreatography, *PTC* percutaneous transhepatic cholangiography, *CRP* C-reactive protein, *POPF* postoperative pancreatic fistula, *BL* biochemical leak

PJ was performed in 7 patients and PG in 16 patients in the PPH C group, and in the control group, the anastomosis was a PJ in 34 patients and a PG in 58 patients. There was no significant difference in the frequencies of the two anastomosis types between the PPH C group and the control group (Table 3). Regarding the number of PG and PJ with confirmed PPH C compared to the total number of patients with PG and PJ in the whole material, the difference was not significant either.

### Complications

In the PPH C group, 6 patients (26%) had no drain amylase leakage postoperatively. Twelve patients (52%) had BL, but all drains were removed during the first postoperative week. Five of 23 patients (22%) had known pancreatic fistulas, grade B POPF, at the time of the PPH C bleed, and they all had the drains left in place since the primary operation.

Two patients had a known biliary leak when PPH C appeared. One of these patients also had a contemporary grade C POPF detected at the time of bleeding. One patient had an undiagnosed biliary leak and no grade C POPF confirmed at the time of PPH C. These three patients with biliary leak had a BL postoperatively.

After the bleeding had emerged, twenty patients could be verified with a grade C POPF, two with leakage in the biliary anastomosis and one with an abdominal infection at the time of PPH C, which were regarded as underlying casual factors.

In the matched control group, 58 patients (63%) did not have any drain amylase leakage postoperatively, 24 (26%) had BL, 4 (4%) had a grade B POPF, and in 6 (7%) data were missing. The missing data were due to participation in a randomized controlled drain study in three of the patients and absence of registration in old medical records in the remaining three. Three patients (4%) had postoperative biliary leak: one was re-operated, and the leakage subsided in the other two after conservative treatment with drains.

The PPH C occurred between postoperative days 6 and 37 (median 16 days: Table 2). Sentinel bleeding, with blood from drains still in place, openings from drains removed, or gastrointestinal bleeding, appeared in 17 (74%) patients before the onset of PPH C. The sites of bleeding were the hepatic artery and branches in 13 patients (57%), the splenic artery in 3 patients (13%), and the branches of the superior mesenteric artery in 1 patient (4%). No definite bleeding site could be defined in the remaining 6 patients (26%).

The 90-day mortality rate in the PPH C group was 35% (8 patients). In the subgroups based on emergency re-operation, angiographic intervention, or a combination performed in attempt to receive bleeding control, the 90-day mortality rate was 42% (5 out of 12 patients), 14% (1 out of 7 patients), and 50% (2 out of 4 patients), respectively (Table 2).

Twelve patients had an emergency re-operation with the purpose to stop the PPH C. During the re-operation, a grade C POPF was verified in eleven of the cases and an abdominal infection in one case. In nine patients, the remnant pancreases were removed due to total detachment from the jejunum or stomach, continues bleeding, or because proper draining could not be accomplished. In two patients, the remnants were left untouched, and only abdominal drains were placed. In one patient, bleeding control was so poor that no further surgery was considered. Five of the twelve patients died, all within 48 h of the re-operation.

Angiographic interventions with embolization or stenting of the bleeding vessels were performed in 11 patients. In four of these patients, the angiographic intervention was performed before or after an emergency re-operation. During the emergency re-operations or shortly after in an additional re-operation, the remnant pancreas was removed in three out of four patients. In this subgroup treated with both operation and angiographic intervention, one patient died shortly after the re-operation and one within 90 days due to postoperative complications. All the patients treated with a combination of angiographic intervention and operations were observed to have grade C POPFs.

Among the seven patients with solely angiographic procedures to attempt to accomplish bleeding control, four were re-operated to remove the remnant pancreas. The purpose of these “rescue” operations was to avoid future bleeds due to pancreatic fistula leakage, when satisfying draining could not be achieved. A grade C POPF was verified perioperatively in all patients who removed the pancreatic remnant. In two of the patients treated with solely angiographic interventions, there were distinct signs that biliary leakage was the casual factor underlying the PPH C and no signs of a grade C POPF. The patients with biliary leakage were treated with percutaneous transhepatic cholangiography (PTC). One of the patients treated with primary angiographic intervention at a regional hospital re-bled and died shortly thereafter. The autopsy could verify a grade C POPF.

In the matched control group, 4 patients (4%) died within 90 days: two due to recurrent disease with massive liver metastasis and multi-organ failure, one due to pulmonary embolism, and one due to liver failure after an unsuccessful venous resection.

The incidence of PPH C among patients in this study was 4.5%.

### Risk factors for PPH C

The univariable analysis of the postoperative factors recording in both the PPH C group and the control group indicated a median CRP POD 2/3 of 210 g/L, CRP POD 2/3 > 180 mg/L, median CRP POD 5/6 of 140 g/L, CRP POD 5/6 > 180 mg/L, and BL and POPF-B to be significant (*p* < 0.05) for PPH C development (Table 3). None of the preoperative or perioperative parameters investigated were found to contribute to an increased risk of PPH C in the univariable analysis (Table 3). The distribution and values of the parameters were similar in the PPH C group and control group.

Of the significant parameters in the univariable analyses, only median CRP POD 5/6 of 140 mg/L was significant for PPH C in the multivariable analysis (Table 4).

**Table 4:** Multivariable conditional logistic regression analysis of predictive factors for postpancreatectomy hemorrhage grade C

	Odds ratio (95% CI)	*p*-value
CRP day 2/3	1.08 (0.99–1.16) *	0.074
CRP day 5/6	1.14 (1.03–1.27) *	0.012

^*^Per 10 units. *CRP* C-reactive protein

## Discussion

This study addresses the issue of identifying patients who have a higher risk of PPH C by trying to verify clinically relevant preoperative, perioperative, and postoperative predictive risk factors. A median CRP POD 5/6 of 140 mg/L was confirmed to be a significant risk factor for PPH C in the multivariable analysis. CRP has a reputation in the literature of indicating postoperative complications. Several studies in pancreatic surgery have determined CRP as an early predictor of septic complications and anastomotic leak [23, 24]. However, most of these studies refer to early CRP values on days 1–3, with cut-offs of 100 mg/L on POD 1, 180 mg/L on POD 2, and 203 mg/L on POD 3 [23, 25, 26]. At this time-point, CT findings might be more difficult to differentiate from postoperative changes [27, 28]. CRP can be increased due to the surgical trauma itself during the first postoperative week. If CRP persists high longer than approximately 3 days, the values seem to be more reliable [29, 30]. Our result with a median CRP POD 5/6 of 140 mg/L is almost concordant with the findings in a recent Dutch study of major complications after PD. To minimize false positive results, they recommend a CRP cut-off of 150 mg/L on POD 5 [24].

All PPH C patients in our study had undetected leaking anastomosis or abdominal infections. Considering the CRP levels, 75% in the PPH C group had CRP above 110 mg/L day 5/6, and only 25% of the patients in the control group had CRP levels above 120 mg/L day 5/6. High postoperative CRP levels can also be due to other conditions like acute pancreatitis or pulmonary embolism.

In the present study, the levels of drain amylase were not clinically relevant for PPH C development. A comparatively large proportion, more than one-quarter, of the patients in the PPH C group, had normal amylase levels in their drains, and about half of the patients presented with only BL before the bleeding emerged. Drains did not eliminate the emergence of PPH C, which is concordant with other drain studies of POPF [12, 13, 31]. Insertion of abdominal drains after pancreatic resection is still a matter of debate, as randomized control studies and meta-analyses have reached contradictory conclusions [12, 14]. Selective drain placements are now advised in many studies [12, 32]. No grade C POPF was known in our study before the PPH C occurred, which is why it is not defined as a *predictive* risk factor for PPH C. However, the connection between POPF and PPH C was distinct, though the vast majority of the patients in the PPH C group had a confirmed grade C POPF during the emergency or rescue operations after presentation of the PPH C. There were no indications of other causal factors of PPH C besides undetected pancreatic or biliary juices or abdominal infection in our study. None of the preoperative or perioperative factors examined were associated with PPH C. The incidence of PPH C after PD was 4.5%, analogous with earlier studies [1, 2].

Different rating systems to try to predict evolvement of POPFs after PD are developed. Two of the most common are postoperative day 1 drain amylase (POD1DA) and the fistula risk score (FRS). They have been found to be equally accurate in predicting clinically relevant POPF [33]. A problem with the POD1DA grading in light of our study is that a notable proportion of the patients, with later observed type C POPFs, had normal POD1DA. Considering FRS, the latest revision defines the score as depending on small duct, soft pancreas, and BMI [34]. The pancreatic texture and the width of the duct were not assessed in the present study, which is a limitation. Considering the pathology report, there is a possibility of a non-negligible proportion of the pancreases having a soft texture. Ampullary and duodenal cancers represented 8 of the 23 specimens in the PPH C group, and 4 of them were grade T1 or Tis. If the tumors are small or situated a definite distance from the pancreatic remnant, the possibility of the parenchyma being soft and unaffected may increase.

Multiple randomized controlled trials and meta-analysis have compared the PJ and PG anastomosis techniques, and it has not been possible to state which of them is superior [35]. Considering the risk of POPF, there has been a slight advantage of performing PG with less leakage [36, 37]. One of the most recent randomized controlled trials with only high-risk anastomosis and externalized stents showed no difference in the primary endpoint POPF but more severe cases of PPH in the PG group [38]. One limitation in that subgroup analysis was that only 5 patients had PPH C. This demonstrates the problems with studies of unusual complications like PPH C. In our study, with 23 patients, no significant differences in the frequency of PPH C could be seen between PJ and PG.

The same concern, with small number of patients, is indicated in the comparatively few studies that have directly addressed risk factors for PPH [2, 5, 6, 39]. Sometimes there are many patients in the original cohort, but in the end, the PPH C subgroup tends to be very small. Although this study, to our knowledge, is the largest with isolated PPH C, it must be recognized that 23 patients are still a small cohort. The low power may influence the probability of significantly identifying small differences in the statistical analyses. The retrospective nature of the study is also a limitation due to the risk of missing data.

Angiographic intervention is well reviewed as an eminent technique managing PPH bleeds [40–42] and has been shown to be superior to re-operation with lower mortality rates [1, 42, 43]. This was confirmed in our study, where the mortality rate was markedly lower in the angiographic intervention subgroup. Sentinel bleeding is important to identify and handle expeditiously. Early angiographic intervention can detect pseudoaneurysms and prevent hemorrhagic shock [44]. In this study, sentinel bleeds appeared in the majority of the patients before the onset of PPH C; in the literature, the number is between 46 and 78% [18, 19]. The hepatic artery and branches were the most common bleeding sites, which is concordant with prior knowledge [18, 45]. The volumes of operations and angiographic interventions have increased at the Sahlgrenska University Hospital over the years, but learning curves of surgeons and interventional radiologists were not analyzed.

Based on this study, one may consider performing a CT scan if CRP is above 140 mg/L on POD day 5 or 6, although an exact CRP cut-off regarding complication risks is difficult to state. Radiological studies confirm CT to be an accurate method for detecting factors predictive of PPH and POPF [46, 47]. Suspicious pancreatic leak with excessive fluid, vascular abnormality, and or intraabdominal abscess can be a sign of surgical complications. Evacuation of the fluid or abscess, preferably by interventional radiology, might reduce the PPH C risk [48].

In conclusion, high postoperative CRP levels are related to the development of PPH C. These findings underline the importance of CRP as a predictor of serious complications after PD surgery and to facilitate clinical decisions postoperatively.
